# Cephalosporin and Ciprofloxacin Resistance in *Salmonella*, Taiwan

**DOI:** 10.3201/eid1106.041153

**Published:** 2005-06

**Authors:** Jing-Jou Yan, Chien-Shun Chiou, Tsai-Ling Yang Lauderdale, Shu-Huei Tsai, Jiunn-Jong Wu

**Affiliations:** * National Cheng Kung University College of Medicine, Tainan, Taiwan;; † Center for Disease Control, Taichung City, Taiwan;; ‡ National Health Research Institutes, Taipei, Taiwan

**Keywords:** salmonella, multidrug resistance, ciprofloxacin, ceftriaxone, CMY-2, β-lactamase

## Abstract

We report the prevalence and characteristics of *Salmonella* strains resistant to ciprofloxacin and extended-spectrum cephalosporins in Taiwan from January to May 2004. All isolates resistant to extended-spectrum cephalosporins carried *bla*_CMY-2_, and all ciprofloxacin-resistant *Salmonella enterica* serotype Choleraesuis isolates were genetically related.

Resistance to extended-spectrum cephalosporins (ESCs) or fluoroquinolones in *Salmonella enterica* has become a global concern (1). ESC resistance in *Salmonella* strains is usually due to the production of plasmid-mediated extended-spectrum β-lactamases (ESBLs) or AmpC β-lactamases, and among these β-lactamases, the CMY-2 AmpC enzyme has been reported most often (1–3). Resistance to fluoroquinolones in *Salmonella* strains is usually due to the accumulation of mutations in the quinolone resistance–determining regions (QRDRs) of DNA gyrase genes (1,4,5). Resistance to both ESCs and fluoroquinolones remains extremely rare in salmonellae.

In Taiwan, increasing resistance to fluoroquinolones and the emergence of CMY-2–producing ESC-resistant strains in salmonellae have been noted (3–6). The emergence of *Salmonella* strains resistant to both ceftriaxone and ciprofloxacin was reported more recently in Taiwan and may pose a serious therapeutic problem (7,8). We conducted the present study to investigate the prevalence and characteristics of *Salmonella* strains resistant to ciprofloxacin and ESCs in Taiwan.

## The Study

From January to May 2004, a total of 600 *Salmonella* isolates from 585 patients were obtained from 5 medical centers and 14 district hospitals throughout Taiwan; these isolates were serotyped with commercial antisera (Difco, Detroit, MI, USA). The 4 most common serotypes of *Salmonella enterica* (Enteritidis, Typhimurium, Stanley, and Choleraesuis) accounted for 66.8% of all isolates. Two isolates were untypeable, and the remainder were typed into 42 serotypes (data not shown), which were each represented by 1 to 23 isolates.

MICs of antimicrobial agents were determined by the agar dilution method (9). Resistance to ciprofloxacin (MIC ≥4 μg/mL) was seen in 50 (8.3%) isolates (Table 1); 20 (3.3%) were resistant (MICs ranging from 8 to >64 μg/mL) to ceftazidime, ceftriaxone, cefotaxime, or aztreonam (Table 2); 6 isolates showed decreased susceptibilities to 1 or 2 of the 4 ESCs (MICs 0.5–2 μg/mL); 10 (1.7%) isolates were resistant to both ciprofloxacin and ESCs. *S*. Choleraesuis had high rates of resistance to ciprofloxacin (84.4%), ESCs (17.8%), and both (17.8%). None of the 26 *Salmonella* isolates with resistance or decreased susceptibility to ESCs produced ESBL, according to the double-disk synergy method (10). Among the 20 ESC-resistant isolates, 10 isolates were ciprofloxacin-resistant, 4 isolates showed decreased susceptibility to ciprofloxacin (MIC 0.25–1 μg/mL) and resistance to nalidixic acid, and 6 isolates were susceptible to ciprofloxacin and nalidixic acid (Table 2). All 20 ESC-resistant isolates were susceptible to cefepime (MIC <0.03 μg/mL) and imipenem (MIC <1 μg/mL), and 17 isolates were resistant to >1 non–β-lactam agent.

**Table 1:** Resistance to ciprofloxacin, extended-spectrum cephalosporins, and both in *Salmonella enterica* serotypes, by region and pulsotype, Taiwan, January–May 2004

Resistance and region*	No. of resistant *S*. *enterica* isolates/no. of total isolates (%)						
	Enteritidis	Typhimurium	Stanley	Choleraesuis	Uncommon serotypes†	All serotypes	Pulsotypes of Choleraesuis isolates (no. of isolates)
Ciprofloxacin resistance	1/161 (0.6)	0/142 (0)	0/53 (0)	38/45 (84.4)	11/199 (5.5)	50/600 (8.3)	
Northern	0/96 (0)	0/38 (0)	0/14 (0)	13/16 (81.3)	6/88 (6.8)	19/252 (7.5)	A (11), D (1), E (1)
Central	1/32 (3.1)	0/34 (0)	0/14 (0)	6/8 (75.0)	4/37 (10.8)	11/125 (8.8)	A (5), C (1)
Southern	0/25 (0)	0/60 (0)	0/24 (0)	18/20 (90.0)	1/65 (1.5)	19/194 (9.8)	A (14), B (1), C (1), F (1), G (1)
Eastern	0/8 (0)	0/10 (0)	0/1 (0)	1/1 (100)	0/9 (0)	1/29 (3.4)	A (1)
ESC resistance	0/161 (0)	0/142 (0)	3/53 (5.7)	8/45 (17.8)	9/199 (4.5)	20/600 (3.3)	
Northern	0/96 (0)	0/38 (0)	1/14 (7.1)	1/16 (6.3)	7/88 (8.0)	9/252 (3.6)	
Central	0/32 (0)	0/34 (0)	1/14 (7.1)	0/8 (0)	1/37 (2.7)	2/125 (0.8)	
Southern	0/25 (0)	0/60 (0)	1/24 (4.2)	7/20 (35.0)	1/65 (1.5)	9/194 (4.6)	
Eastern	0/8 (0)	0/10 (0)	0/1 (0)	0/1 (0)	0/9 (0)	0/29 (0)	
Ciprofloxacin and ESC resistance	0/161 (0)	0/142 (0)	0/53 (0)	8/45 (17.8)	2/199 (1.0)	10/600 (1.7)	
Northern	0/96 (0)	0/38 (0)	0/14 (0)	1/16 (6.3)	1/88 (1.1)	2/252 (0.8)	E (1)
Central	0/32 (0)	0/34 (0)	0/14 (0)	0/8 (0)	1/37 (2.7)	1/125 (0.8)	
Southern	0/25 (0)	0/60 (0)	0/24 (0)	7/20 (35.0)	0/65 (0)	7/194 (3.6)	A (4), C (1), F (1), G (1)
Eastern	0/8 (0)	0/10 (0)	0/1 (0)	0/1 (0)	0/9 (0)	0/29 (0)	

**Table 2:** Characteristics of 20 *Salmonella* isolates resistant to extended-spectrum cephalosporins

Serotype	Specimen type	pI (s)	Resistance pattern*	*gyrA* at position†			
				83 (TCC [Ser])	87 (GAC [Asp])	*parC* at position 80 (AGC [Ser])‡	Isolate (restriction pattern of transferred *bla*_CMY-2_+ plasmid)§
Albany	Urine	9.0	Am ESC Fx Cm Na Sxt Tc†	–	AAC (Asn)	–	SA04.028 (C)
Cairo	Stool	9.0, 5.4	Am ESC Fx Cm Na Gm Km Sxt Tc†	TTC (Phe)	–	–	NC04.001 (H1), NC04.002 (H1), NC04.003 (H1)
	Urine	9.0	Am ESC Fx Cm Cp Na Sxt Tc	TTC (Phe)	GGC (Gly)	AAC (Arg)	NC04.004 (H2)
Chester	Stool	9.0	Am ESC Fx	–	–	–	NG04.016 (G)
Choleraesuis	Wound	9.0, 5.4	Am ESC Fx Cm Cp Na Gm Km Sxt Tc	TTC (Phe)	AAC (Asn)	ATC (Ile)	NL04.050 (B)
	Blood	9.0, 5.4	Am ESC Fx Cm Cp Na Gm Tc	TTC (Phe)	AAC (Asn)	ATC (Ile)	SB04.003 (A)
	Blood	9.0, 5.4	Am ESC Fx Cm Cp Na Gm Km Sxt Tc	TTC (Phe)	AAC (Asn)	ATC (Ile)	SE04.005 (F), SG04.060
	Blood	9.0, 5.4	Am ESC Fx Cm Cp Na Gm Km Sxt	TTC (Phe)	AAC (Asn)	ATC (Ile)	SG04.039 (E1), SG04.086
	Joint fluid	9.0, 5.4	Am ESC Fx Cm Cp Na Gm Km Sxt Tc	TTC (Phe)	AAC (Asn)	ATC (Ile)	SG04.042 (E2), SG04.047 (E4)
Kaduna	Tissue	9.0, 5.4	Am ESC Fx Cm Cp Na Sxt Tc	TTC (Phe)	AAC (Asn)	ATC (Ile)	CE04.015 (I)
Saintpaul	Stool	9.0	Am ESC Fx Gm	–	–	–	NG04.011 (G), NG04.018 (G)
Stanley	Stool	9.0, 5.4	Am ESC Fx Cm Sxt Tc	–	–	–	CG04.039 (D)
	Stool	9.0	Am ESC Fx Cm Sxt Tc	–	–	–	NB04.022 (A), SE04.006 (E3)

*Am, ampicillin; ESC, extended-spectrum cephalosporins; Fx, cefoxitin; Cm, chloramphenicol, Cp, ciprofloxacin; Na, nalidixic acid; Gm, gentamicin; Km, kanamycin; Sxt, trimethoprim-sulfamethoxazole; Tc, tetracycline. †The *S*. Albany isolate and the 3 *S*. Cairo isolates showed decreased susceptibilities to ciprofloxacin (MIC 0.25–1 μg/mL). ‡Nucleotide and amino acid changes at the QRDRs of *gyrA* and *parC*. –, no alterations in the genes. §For each isolate, the first letter indicates region (C, central region; N, northern region; S, southern region), and the second letter represents hospital. Isolates NC04.001, NC04.002, and NC04.003 were from the same patients; all other isolates were from different patients.

All 20 ESC-resistant isolates expressed a β-lactamase of pI 9.0 by isoelectric focusing (3,11); 11 of these isolates expressed an additional pI 5.4 β-lactamase (Table 2). *bla*_CMY-2_ was detected in all ESC-resistant isolates. *bla*_TEM-1_ was detected in the 11 isolates with the pI 5.4 β-lactamase by polymerase chain reaction (PCR) and sequence analyses with the primers for the entire *bla*_TEM_-related and *bla*_CMY-2_-related structural genes (2,3).

The QRDR sequences of *gyrA*, *gyrB*, *parC*, and *parE* of the 20 ESC-resistant *Salmonella* isolates were determined by PCR and sequence analyses (5). All 10 ciprofloxacin-resistant isolates showed 2 mutations at the Ser-83 and Asp-87 codons in *gyrA* and a single mutation at the Ser-80 codon in *parC* (Table 2). Four isolates with decreased susceptibility to ciprofloxacin had a single mutation at either the Ser-83 or the Asp-87 codon in *gyrA*. All 20 ESC-resistant isolates showed no mutations in the QRDRs of *gyrB* and *parE*.

**Figure 1:**
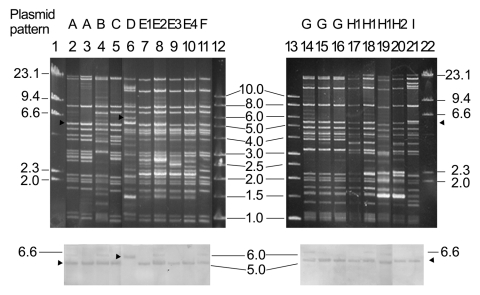
*Eco*RI restriction patterns of transferred CMY-2–encoding plasmids of 18 *Salmonella* isolates. The result of the hybridization assay with the *bla*_CMY-2_ probe labeled with digoxigenin (Roche Molecular Biochemicals, Mannheim, Germany) is shown below the gel, and arrowheads indicate the locations of the restriction fragments that were hybridized. Lanes 2–21, plasmids from transconjugants of *Salmonella* isolates NB04.022, SB04.003, NL04.050, SA04.028, CG04.039, SG04.039, SG04.042, SE04.006, SG04.047, SE04.005, NG04.011, NG04.016, NG04.018, NC04.001, NC04.002, NC04.003, NC04.004, and CE04.015; lanes 1 and 22, molecular marker II (Roche Molecular Biochemicals); lanes 12 and 13, a 1-kb molecular marker (Promega Co., Madison, WI, USA).

ESC resistance was transferred from 18 of the 20 ESC-resistant *Salmonella* isolates to *Escherichia coli* C600 in the liquid mating-out assay (3,12). All transconjugants showed decreased susceptibilities to the 4 ESCs tested (MICs 16–64 μg/mL) and cefoxitin (MIC 64–128 μg/mL) and were susceptible to all non–β-lactam agents tested. A pI 9.0 vz β-lactamase and *bla*_CMY-2_ were detected by isoelectric focusing and PCR assays, respectively, in all transconjugants. Restricted by the endonuclease *Eco*RI, the 18 transferred plasmids produced 9 major restriction patterns (Figure 1 and Table 2). Patterns E and I were further divided into 4 and 2 subtypes, respectively. *bla*_CMY-2_ on the transferred plasmids was demonstrated by Southern hybridization with the *bla*_CMY-2_ probe.

**Figure 2:**
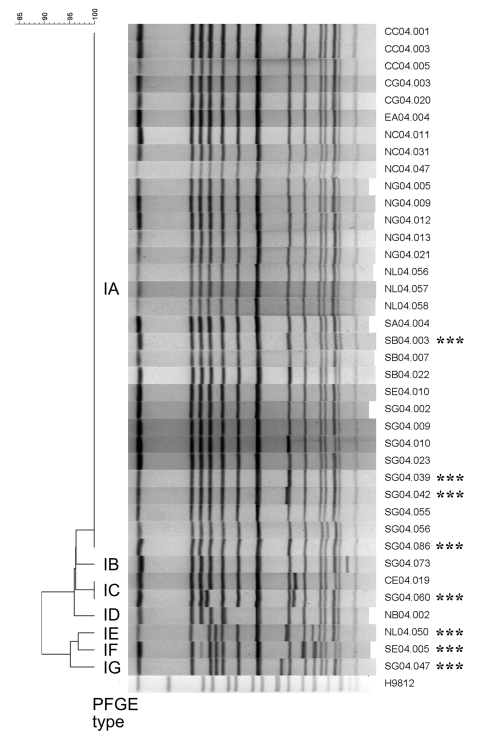
Dendrogram (left) obtained from cluster analysis of *Xba*I-generated macrorestriction patterns of 38 ciprofloxacin-resistant *Salmonella enterica* serotype Choleraesuis (right). Asterisks indicate extended-spectrum cephalosporin-resistant isolates. A percent scale of similarity is shown above the dendrogram. Pulsed-field gel electrophoresis (PFGE) types are shown between the gel and the dendrogram. H9812, *S. enterica* serotype Braenderup strain H9812, which was used as reference size marker.

The 38 ciprofloxacin-resistant *S*. Choleraesuis isolates were genotyped by pulsed-field gel electrophoresis on a CHEF Mapper apparatus (Bio-Rad Laboratories, Hercules, CA, USA) according to the PulseNet protocol (13). Banding patterns generated by *Xba*I restriction were compared with BioNumerics software (Applied Maths, Kortrijk, Belgium). The 38 isolates showed a close relationship (Dice correlation coefficient of 90%) and had only 1 pulsotype, based on Tenover criteria (Figure 2) (14). The pulsotype was divided into 7 pulsosubtypes, among which were 1–4 band differences. Five ESC-resistant isolates displayed the same pulsosubtypes (IA or IC) as ESC-susceptible isolates (Table 1 and Figure 2).

## Conclusions

We describe the prevalence of resistance to ciprofloxacin and ESCs among salmonellae isolated from January to May 2004 in Taiwan. We found widespread resistance of *Salmonella* isolates to both ESCs and ciprofloxacin; high prevalence of resistance to ciprofloxacin, ESCs, and both in *S*. Choleraesuis; and widespread prevalence of CMY-2–producing *Salmonella* isolates of various serotypes in Taiwan.

The prevalence of *Salmonella* isolates resistant to both ceftriaxone and ciprofloxacin may pose a therapeutic problem. CMY-2 is one of the AmpC enzymes, which are usually less active against cefepime and cefpirome than ESBLs (15). Accordingly, we have used cefepime to successfully treat several patients infected with CMY-2–producing and ciprofloxacin-resistant *S*. Choleraesuis (8). Therefore, AmpC-producing strains should be differentiated from ESBL-producing strains by phenotypic or genotypic methods when ESC-resistant *Salmonella* strains are isolated in the clinical microbiology laboratory (15).

The ciprofloxacin-resistant rate in *S*. Choleraesuis in Taiwan has been >60% since 2001; the high prevalence was mainly due to clonal spread of resistant strains (4–6). The ciprofloxacin-resistant rate in *S*. Choleraesuis in this report (84.4%) was higher than those reported previously (≤70%) (4–6). *bla*_CMY-2_ in *Salmonella* in Taiwan was first reported in 2 *S*. Typhimurium strains isolated in 2000 (3). The first reported *S*. Choleraesuis strain with *bla*_CMY-2_ was a ciprofloxacin-resistant strain isolated in 2002 (7). All our 38 ciprofloxacin-resistant *S*. Choleraesuis isolates, including 8 ESC-resistant isolates, were genetically related. Moreover, we found possibly unrelated *bla*_CMY-2_-positive plasmids (lanes 3, 4, 7, 8, 10, and 11 in Figure 1) among closely related isolates (Figure 2). These data together suggest that the development and rapidly increasing prevalence of ESC and ciprofloxacin resistance in *S*. Choleraesuis in Taiwan might result from the extremely high prevalence of ciprofloxacin resistance followed by the horizontal transfer of *bla*_CMY-2_ into ciprofloxacin-resistant epidemic strains rather than from the spread of a clone that had been resistant to ciprofloxacin and ESCs.

All our ciprofloxacin-resistant *Salmonella* isolates tested had mutations in the QRDRs of *gyrA* and *par*, a finding consistent with previously reported results (1,4,5). The rates of ciprofloxacin resistance in the 3 most common serotypes, Enteritidis, Typhimurium, and Stanley, remained very low (0%–0.6%). Six of 11 ciprofloxacin-resistant isolates in the group of uncommon serotypes belonged to serotype Schwarzengrund and accounted for 42.9% of all serotype Schwarzengrund isolates. Thus, the high rate (5.5%) of ciprofloxacin resistance in this group was in part due to the high prevalence of ciprofloxacin resistance in serotype Schwarzengrund.

## References

[1] Su LH, Chiu CH, Chu C, Ou JT. Antimicrobial resistance in nontyphoid *Salmonella* serotypes: a global challenge. Clin Infect Dis. 2004;39:546–51. 10.1086/422726 15356819

[2] Winokur PL, Brueggemann A, Desalvo DL, Hoffmann L, Apley MD, Uhlenhopp EK, Animal and human multidrug-resistant, cephalosporin-resistant *Salmonella* isolates expressing a plasmid-mediated CMY-2 AmpC β-lactamase. Antimicrob Agents Chemother. 2000;44:2777–83. 10.1128/AAC.44.10.2777-2783.2000 10991860 PMC90151

[3] Yan JJ, Ko WC, Chiu CH, Tsai SH, Wu HM, Wu JJ. Emergence of ceftriaxone-resistant *Salmonella* isolates and the rapid spread of plasmid-encoded CMY-2-like cephalosporinase, Taiwan. Emerg Infect Dis. 2003;9:323–8. 12643826 10.3201/eid0903.010410 PMC2958529

[4] Chiu CH, Wu TL, Su LH, Chu C, Chia JH, Kuo AJ, The emergence in Taiwan of fluoroquinolone resistance in *Salmonella enterica* serotype Choleraesuis. N Engl J Med. 2002;346:413–9. 10.1056/NEJMoa012261 11832529

[5] Hsueh PR, Teng LJ, Tseng SP, Chang CF, Wan JH, Yan JJ, Ciprofloxacin-resistant *Salmonella enterica* Typhimurium and Choleraesuis from pigs to humans, Taiwan. Emerg Infect Dis. 2004;10:60–8. 15078598 10.3201/eid1001.030171 PMC3322755

[6] Chiu CH, Wu TL, Su LH, Liu JW, Chu C. Fluoroquinolone resistance in *Salmonella enterica* serotype Choleraesuis, Taiwan, 2000–2003. Emerg Infect Dis. 2004;10:1674–6. 15498176 10.3201/eid1009.030596 PMC3320291

[7] Chiu CH, Su LH, Chu C, Chia JH, Wu Tl, Lin TY, et al. Isolation of *Salmonella enterica* serotype Choleraesuis resistant to ceftriaxone and ciprofloxacin. Lancet. 2004;363:1285–6. 10.1016/S0140-6736(04)16003-0 15094275

[8] Ko WC, Yan JJ, Yu WL, Lee HC, Lee NY, Wang LR, A new therapeutic challenge for old pathogens: invasive community-acquired infections caused by ceftriaxone- and ciprofloxacin-resistant *Salmonella enterica* serotype Choleraesuis. Clin Infect Dis. 2005;40:315–8. 10.1086/426593 15655754

[9] NCCLS. Methods for dilution antimicrobial susceptibility tests for bacteria that grow aerobically; approved standard. 6th ed. M7-A6. Wayne (PA): The Committee; 2003.

[10] Jarlier V, Nicolas MH, Fournier G, Philippon A. Extended broad-spectrum β-lactamases conferring transferable resistance to newer β-lactam agents in *Enterobacteriaceae*: hospital prevalence and susceptibility patterns. Rev Infect Dis. 1988;10:867–78. 10.1093/clinids/10.4.867 3263690

[11] Matthew M, Harris M, Marshall MJ, Rose GW. The use of analytical isoelectric focusing for detection and identification of β-lactamases. J Gen Microbiol. 1975;88:169–78. 807678 10.1099/00221287-88-1-169

[12] Provence DL, Curtiss R III. Gene transfer in gram-negative bacteria. In: Gerhardt P, Murray RGE, Wood WA, Krieg NR, editors. Methods for general and molecular bacteriology. Washington: American Society for Microbiology; 1994. p. 319–47.

[13] Graves LM, Swaminathan B. PulseNet standardized protocol for subtyping *Listeria monocytogenes* by macrorestriction and pulsed-field gel electrophoresis. Int J Food Microbiol. 2001;65:55–62. 10.1016/S0168-1605(00)00501-8 11322701

[14] Tenover FC, Arbeit R, Goering RV, Mickelsen PA, Murray BE, Persing DH, Interpreting chromosomal DNA restriction patterns produced by pulsed-field gel electrophoresis: criteria for bacterial strain typing. J Clin Microbiol. 1995;33:2233–9. 7494007 10.1128/jcm.33.9.2233-2239.1995 PMC228385

[15] Philippon A, Arlet G, Jacoby GA. Plasmid-determined AmpC-type β-lactamases. Antimicrob Agents Chemother. 2002;46:1–11. 10.1128/AAC.46.1.1-11.2002 11751104 PMC126993

